# Can novel technologies improve breast conserving surgery?

**DOI:** 10.1186/s13058-018-1016-9

**Published:** 2018-08-03

**Authors:** Brian W. Pogue

**Affiliations:** 1 Center for Imaging Medicine, Thayer School of Engineering at Dartmouth, Hanover, USA; 2 Department of Surgery, Geisel School of Medicine at Dartmouth, Hanover, USA

The practice of breast conserving surgery has been transformative for management of women’s breast cancer [1], and yet the current practice remains in a situation where approximately one-third of all patients have incomplete surgical resection. This is measured by the finding of clear margins on the surgical specimen, as measured by pathology sampling. This is a very active area of professional debate and research study [2], and the solutions are not as obvious as one might guess. Still, reviews of the status of the field suggest that technical solutions should be available to help mitigate this issue [3], and the tools for molecular phenotyping of tissues need to be deployed if they can provide rapid, specific diagnoses.

The paper by Shipp et al. [4] published in this issue presents one of the most promising technologies to molecular fingerprint a tissue, through Raman spectroscopy. In particular, the hypothesis in this paper is that, through a combination of fluorescence imaging to map a tissue macroscopically (4 × 6.5 cm^2^) and Raman spectroscopy to fingerprint several microscopic areas, the strengths of each might be combined into a better overall tool for a fast, surgical environment. Raman spectroscopy has incredible potential for specificity because of the nature of generating dozens to hundreds of molecular-specific vibrational bands from each sample. Yet, its limitations of long acquisition time, low signal to noise, and some non-specific peaks have hampered the translation of this from individual investigator studies into multicenter studies. However, even though optimized and ‘fast’ acquisitions systems have been presented for over two decades [5, 6], discoveries in the past few years of how to further maximize the signal and thereby improve the speed of acquisition have been important [7, 8]. Additionally, being able to capture Raman signals in a noisy light environment has been an issue, which can be partially solved by collection geometry and partially solved by smart algorithms [9]. Similarly, improved data processing methods with accurate and robust discriminant analysis are important, as presented in the work of Shipp et al.

One of the strengths of their study is the dual phased approach to validating the system, where the spectral training was done on frozen tissues with known pathology (*n* = 91 frozen samples) and this trained algorithm was then utilized on a separate set of tissues (*n* = 70 frozen samples). Once validated this way, in the second phase they next applied the approach to image and classify freshly resected specimens (*n* = 51), achieving 100% sensitivity and 80% specificity.

While this study is promising, there are still limitations which need to be addressed in future research work; these are in the areas of 1) sufficient pathobiology sampling, 2) hardware optimization, and 3) software optimization. All of these will need to be solved prior to any real commercial translation [10]. The study used a good variation of tumor and normal/benign tissue subtypes, but the large number of these illustrates the need for higher sample numbers, because with three types of carcinoma and tumors, DCIS, three benign tumors, inflammation, and four types of normal tissues the range expected in sampling is high, and even with 51 samples this range cannot be covered adequately with sufficient statistics. Systems with this potential specificity will need hundreds to thousands of tissues to gain robust stable classification of undiagnosed tissues. In the area of hardware, the acquisition time of 12–14 min is too long to be useful for most surgery applications, and so improvements in automation of acquisition and optimization are required. The current approach of imaging through a window is likely essential for automation, and so this places some major constraints on the geometry and applications possible. Finally, as systems emerge which might go into multicenter trials or regulatory clearance, factors that affect the algorithms and need for calibration and retraining will require careful thought. These post-processing and classification algorithms can be non-linear and saturable in nature, and so careful attention to making them as robust as possible will be essential, and this can only be verified with the largest possible data sets. It is possible that future banks of known classified tissue spectra could be used to test and calibrate systems with the appropriate controls on their use.

Still, despite these limitations, this single site study presents two major steps forward in the ability to do molecular fingerprinting of surgical tissues, by first showing that a combination of optical imaging tools can speed up the choice of tissues to sample, and secondly that analysis by Raman spectroscopy appears stable from training on frozen tissues to freshly resected tissues in the breast.
